# 

**Published:** 1840-12

**Authors:** 


### THE
### WESTERN JOURNAL
### OF
### MEDICINE AND SURGERY:

EDITED BY

DANIEL DRAKE, M. D.

AND

LUNSFORD P. YANDELL, M. D.

PROFESSORS IN THE LOUISVILLE MEDICAL INSTITUTE.

---

NO. XII.—DECEMBER, 1840.

VOL. II.—NO. VI.

---

LOUISVILLE, KY.

PUBLISHED MONTHLY, BY PRENTICE & WEISSINGER,

PROPRIETORS AND PRINTERS.

1840.

*This Number contains 4 sheets—Postage under 100 miles 6 cents, over 100 miles 10 cents.*

